# Developing a Stakeholder‐Informed Multilevel Framework for Skin Cancer Prevention: A Qualitative Study of Barriers, Policy Priorities and Implementation Strategies

**DOI:** 10.1155/jskc/6064093

**Published:** 2026-11-08

**Authors:** Masoumeh Gholizadeh, Mehran Sardareh, Amin Ghanbari, Safoura Pourreza, Rona Bahreini

**Affiliations:** ^1^ Hematology and Oncology Research Center Tabriz University of Medical Sciences Tabriz Iran tbzmed.ac.ir; ^2^ Iranian Center of Excellence in Health Management (IceHM) Tabriz University of Medical Sciences Tabriz Iran tbzmed.ac.ir; ^3^ Medical Philosophy and History Research Center Tabriz University of Medical Sciences Tabriz Iran tbzmed.ac.ir; ^4^ Tabriz Health Services Management Research Center Tabriz University of Medical Sciences Tabriz Iran tbzmed.ac.ir; ^5^ Student Research Committee Tabriz University of Medical Sciences Tabriz Iran tbzmed.ac.ir

**Keywords:** health policy, implementation science, prevention, public health, qualitative research, skin cancer, stakeholder engagement, thematic analysis

## Abstract

**Background:**

Skin cancer is the most common malignancy worldwide, and its incidence continues to increase despite substantial advances in diagnosis and treatment. While effective preventive measures are well established, implementation of coordinated prevention strategies remains fragmented across healthcare systems. Existing interventions often operate independently, with limited collaboration among healthcare providers, policymakers, educators, employers and communities. Consequently, there is an urgent need for an evidence‐informed framework that integrates stakeholder perspectives into practical prevention strategies.

**Objective:**

To explore stakeholders’ perceptions of barriers to skin cancer prevention and develop a stakeholder‐informed implementation framework to support coordinated prevention policies.

**Methods:**

A qualitative study using semistructured interviews was conducted with purposively selected stakeholders from clinical practice, public health, health policy, occupational health and academia. Interviews were transcribed verbatim and analysed using Braun and Clarke’s six‐phase reflexive thematic analysis. Trustworthiness was enhanced through investigator triangulation, synthesised member checking and an audit trail. Reporting followed the Consolidated Criteria for Reporting Qualitative Research (COREQ).

**Results:**

Stakeholders identified interconnected barriers operating across individual, organisational, healthcare system, environmental and policy levels. Key challenges included inadequate public awareness, fragmented prevention programmers, insufficient interpectoral collaboration, limited occupational protection and weak policy commitment. Participants proposed integrated solutions encompassing public education, environmental modification, workplace protection, strengthened primary healthcare, targeted screening and governmental leadership. These findings informed the development of a multilevel implementation framework linking barriers to actionable prevention strategies.

**Conclusion:**

Effective skin cancer prevention requires coordinated action extending beyond individual behavioural change. The stakeholder‐informed framework developed in this study provides an evidence‐based roadmap for integrating prevention into healthcare systems, educational institutions, workplaces and public policy. Future implementation studies should evaluate the feasibility and effectiveness of this framework across diverse healthcare settings.

## 1. Introduction

Skin cancer is the most common malignancy worldwide and represents a major and growing public health challenge. The global burden of both melanoma and nonmelanoma skin cancers has increased steadily over recent decades due to population ageing, cumulative ultraviolet (UV) radiation exposure, behavioural changes, occupational risks and environmental factors. Recent global cancer estimates indicate that skin cancers constitute a substantial proportion of all newly diagnosed malignancies, with melanoma continuing to contribute disproportionately to mortality despite representing a smaller proportion of total skin cancer cases [1, 2]. Although mortality associated with most nonmelanoma skin cancers remains relatively low, advanced melanoma continues to impose considerable disease‐specific mortality, treatment costs and healthcare burden [3]. Consequently, skin cancer prevention has become an international public health priority requiring coordinated action across healthcare systems, communities and policy environments [4].

Significant advances have transformed the management of advanced skin cancer, particularly through the introduction of immune checkpoint inhibitors targeting the programmed death‐1 (PD‐1) pathway and other immune regulatory mechanisms. Contemporary clinical evidence demonstrates that immunotherapy has substantially improved survival outcomes among patients with advanced melanoma and other advanced cutaneous malignancies [5, 6]. Furthermore, recent comparative effectiveness studies suggest that first‐line immunotherapy may provide superior long‐term survival outcomes compared with targeted therapies in selected patient populations, reinforcing its central role in modern melanoma management [7]. Nevertheless, these therapeutic advances primarily benefit individuals with established disease and remain associated with substantial costs, treatment‐related toxicities and unequal accessibility across healthcare systems. Therefore, prevention and early detection remain the most effective and economically sustainable strategies for reducing the future burden of skin cancer [8].

A substantial proportion of skin cancers are preventable through evidence‐based interventions, including reduction of UV exposure, adoption of sun‐protective behaviours, occupational protection measures, environmental modifications and early detection programmes [9, 10]. Evidence from randomised trials and population‐based studies indicates that consistent sunscreen use and comprehensive photoprotection strategies can reduce the occurrence of certain skin cancers, particularly melanoma and actinic damage [11]. However, the effectiveness of prevention programmes depends not only on individual behaviour but also on broader social, organisational and policy contexts. Occupational exposure remains a major concern, particularly among outdoor workers who experience prolonged cumulative UV exposure and often face structural barriers to adopting protective behaviours [12]. Although public awareness campaigns, school‐based interventions, workplace programmes and screening initiatives have demonstrated varying levels of effectiveness, their implementation remains inconsistent across settings [13].

Implementation science increasingly recognises that sustainable public health interventions depend on meaningful collaboration among multiple stakeholders throughout programme development, implementation, adaptation and evaluation [14]. Skin cancer prevention represents a complex public health challenge requiring coordinated engagement among clinicians, public health professionals, policymakers, educators, employers, occupational health specialists, environmental planners, media organisations and communities [15]. Importantly, patients should be recognised as active partners in identifying prevention priorities and shaping future research agendas rather than passive recipients of healthcare services. Recent evidence exploring patient priorities in melanoma research has highlighted prevention, early detection and improved access to preventive strategies as key areas requiring further investigation, emphasising the importance of incorporating patient perspectives into prevention policy development [16].

Despite substantial advances in skin cancer prevention research, important evidence gaps remain regarding the translation of evidence‐based interventions into sustainable population‐level practice. Previous investigations have predominantly evaluated individual preventive strategies, including educational campaigns, sunscreen promotion, workplace protection initiatives and screening programmes, as isolated interventions. Although these approaches have demonstrated variable effectiveness, their impact is frequently limited by inconsistent implementation, inadequate integration within health systems and insufficient consideration of contextual determinants influencing preventive behaviours [17, 18]. Consequently, contemporary prevention research increasingly emphasises the need to move beyond intervention effectiveness alone and investigate the mechanisms through which preventive strategies can be successfully adopted, implemented and sustained within real‐world settings [19].

Existing prevention programmes may operate across multiple sectors and organisational settings, creating challenges related to coordination, fragmentation and the alignment of responsibilities and resources. Evidence from population health research has identified siloed service delivery, competing priorities, short‐term funding and political cycles, and weak interorganisational coordination as important barriers to sustaining prevention programmes. Contemporary implementation science further emphasises that the successful implementation and sustainability of complex health interventions depend on the interaction between intervention characteristics, organisational capacity, leadership, available resources, interorganisational relationships and the broader sociopolitical and policy context [14, 20]. Therefore, improving skin cancer prevention requires the development of integrated implementation frameworks capable of connecting stakeholder experiences with practical strategies adaptable to diverse healthcare and social contexts.

The current literature on skin cancer prevention has largely focused on epidemiological patterns, risk factors, ultraviolet (UV) exposure, preventive behaviours and the effectiveness of individual‐ and population‐level interventions. Recent evidence syntheses indicate that prevention research remains strongly oriented toward evaluating intervention effects and preventive strategies, whereas comparatively less attention has been devoted to understanding the organisational, institutional and policy contexts that shape their adoption, implementation and sustainability in routine practice. This gap is particularly relevant because successful prevention requires more than demonstrating intervention effectiveness; it also depends on understanding the contextual and system‐level conditions that facilitate or constrain implementation across real‐world settings [21].

Quantitative approaches are essential for estimating disease burden and identifying associations; however, they provide limited insights into why prevention strategies succeed or fail within specific healthcare environments. Implementation challenges are inherently context‐dependent and shaped by professional roles, organisational structures, resource availability, social norms and governance mechanisms [22]. Qualitative research therefore provides a valuable methodological approach for exploring these complex interactions and generating contextually relevant explanations for implementation barriers and facilitators.

The present study is conceptually informed by contemporary principles of implementation science, which emphasise the systematic translation of evidence into routine healthcare practice and public health settings. Successful implementation depends not only on the effectiveness of an intervention but also on contextual conditions, organisational readiness and capacity, stakeholder engagement, appropriate implementation strategies and the ability to sustain changes over time. Recent implementation research highlights the importance of understanding context at individual, organisational and broader system levels, as well as tailoring implementation strategies to local needs and conditions [22, 23]. Frameworks such as the Consolidated Framework for Implementation Research (CFIR) provide structured approaches for identifying multilevel determinants affecting intervention adoption and implementation, including intervention characteristics, inner organisational settings, external environments, individual characteristics and implementation processes [22].

This perspective aligns closely with ecological models of health promotion, which emphasise that preventive behaviours emerge from dynamic interactions between individuals and their surrounding social, environmental, organisational and policy contexts [24]. Accordingly, approaches focusing exclusively on individual knowledge or motivation are unlikely to achieve sustained population‐level improvements unless supported by enabling environments, institutional commitment, occupational protection systems and supportive public policies [25]. In the context of skin cancer prevention, this suggests that effective strategies should integrate individual education with organisational interventions, environmental modifications, regulatory mechanisms and coordinated governance structures.

Meaningful stakeholder engagement has increasingly been recognised as an important component of health policy development and implementation research. Engagement with policymakers, implementers and other relevant stakeholders can facilitate coproduction, contextualisation of evidence and translation of research findings into policy and practice. Stakeholders can also contribute experiential and contextual knowledge regarding implementation barriers, resource constraints, feasibility, local priorities and opportunities for adaptation that may be less visible in conventional epidemiological or effectiveness‐focused research [26, 27]. Evidence indicates that involving stakeholders throughout intervention design and implementation enhances acceptability, relevance, ownership and sustainability of public health initiatives [26]. Therefore, stakeholder engagement should not be regarded as a one‐time consultation process but rather as an iterative mechanism through which evidence is interpreted, adapted and translated into feasible prevention strategies.

In skin cancer prevention, relevant stakeholders extend beyond healthcare professionals to include public health practitioners, policymakers, educators, occupational health specialists, employers, environmental planners, community organisations, media representatives and patients. These groups operate at different levels of the prevention system and encounter distinct challenges related to implementation, communication, resource allocation and policy enforcement. Exploring areas of convergence and divergence among these perspectives can provide critical insights for developing prevention programmes that are evidence‐informed, context‐sensitive, equitable and sustainable.

Qualitative research is particularly valuable for investigating complex implementation challenges because it enables in‐depth examination of experiences, perceptions, organisational processes and contextual influences that may be difficult to capture through quantitative approaches alone. Whereas quantitative studies can estimate the prevalence of preventive behaviours, assess intervention effects and examine statistical associations between exposures and outcomes, qualitative methods can provide complementary insight into how interventions are experienced, interpreted, adapted and implemented within specific organisational and social contexts. Such approaches can also help elucidate the contextual and process‐related factors that shape adoption, implementation, adaptation and sustainability in real‐world settings [28, 29].

Semistructured interviews represent an effective qualitative approach for exploring stakeholder perspectives because they allow participants to articulate professional experiences, perceived barriers, contextual challenges and potential solutions while maintaining sufficient consistency across interviews. The flexibility of this approach enables researchers to investigate unexpected issues emerging during data collection and to capture the complexity of multidisciplinary prevention systems [30]. Through systematic thematic analysis, individual narratives can be synthesised into higher‐order patterns and conceptual categories that support the development of explanatory frameworks for policy, programme design and implementation strategies [31, 32].

The application of thematic analysis is particularly valuable in implementation‐oriented research because it provides a structured yet flexible method for identifying common patterns across diverse stakeholder groups while preserving the contextual meaning of participants’ experiences. Reflexive thematic analysis allows researchers to move beyond simple categorisation of responses and develop interpretative insights regarding relationships among individual behaviours, organisational processes, institutional structures and broader policy environments [31, 32].

Against this background, the present study aimed to explore stakeholders’ perceptions of barriers affecting effective skin cancer prevention and to identify practical strategies capable of strengthening prevention across multiple sectors of the healthcare system. Rather than focusing solely on individual behavioural determinants, this study adopted an implementation‐oriented perspective to examine how organisational, healthcare, environmental and policy‐level factors interact to influence prevention effectiveness.

Specifically, this study sought to

1. Explore stakeholder perceptions regarding the principal barriers limiting effective skin cancer prevention.
2. Identify organisational, healthcare, environmental and policy‐level strategies for improving prevention programmes.
3. Examine areas of consensus and divergence among stakeholders from different professional backgrounds.
4. Develop a stakeholder‐informed, multilevel implementation framework capable of translating qualitative findings into actionable public health policy.

By integrating diverse stakeholder perspectives into a coherent conceptual framework, this study contributes to the growing literature on implementation science, health systems strengthening and evidence‐informed public health policy. The proposed framework aims to support researchers, clinicians, policymakers and public health practitioners in designing more coordinated, equitable and sustainable skin cancer prevention programmes.

## 2. Method

### 2.1. Study Design

This qualitative study employed an interpretive descriptive design using semistructured individual interviews to explore stakeholders’ perspectives on barriers to skin cancer prevention and potential implementation strategies. An interpretive qualitative approach was selected because the study sought to understand how professionals with diverse roles perceive complex organisational, behavioural and policy‐related challenges that cannot be adequately examined using quantitative methods.

The study design, conduct and reporting were guided by the Consolidated Criteria for Reporting Qualitative Research (COREQ) [33] to ensure transparency and methodological rigor throughout the research process.

### 2.2. Study Setting

The study was conducted in collaboration with the Hematology and Oncology Research Center and the Iranian Center of Excellence in Health Management at Tabriz University of Medical Sciences, Iran. Participants represented organisations involved in clinical care, public health, health policy, academic research and health service management. This multidisciplinary setting enabled exploration of skin cancer prevention from multiple professional perspectives rather than a single institutional viewpoint.

### 2.3. Participants and Sampling

A purposive maximum variation sampling strategy was employed to recruit information‐rich participants capable of providing diverse insights into skin cancer prevention policy and implementation.

Potential participants were identified through professional networks, institutional directories and recommendations from senior researchers familiar with national skin cancer prevention activities. Maximum variation was sought regarding

• Professional discipline;
• Years of experience;
• Academic background;
• Organisational responsibilities; and
• Involvement in skin cancer prevention, health promotion or health policy.

### 2.4. Inclusion Criteria

Participants were eligible if they

• Possessed at least 5 years of professional experience in a relevant field;
• Had direct experience in clinical practice, health policy, dermatology, oncology, occupational health, public health or health management;
• Were willing to participate in an audio‐recorded interview; and
• Were able to communicate their experiences in Persian.

Individuals without relevant professional experience or unwilling to provide informed consent were excluded.

### 2.5. Recruitment Procedure

Eligible individuals received an invitation describing the study objectives, voluntary nature of participation, confidentiality procedures and expected interview duration. Those expressing interest were contacted by the research team to arrange an interview at a mutually convenient time and location.

Written informed consent was obtained before each interview.

### 2.6. Data Collection

Data were collected through semistructured face‐to‐face interviews conducted by trained qualitative researchers with prior experience in health policy research.

The interview guide was developed following a review of the international literature on skin cancer prevention and implementation research and was refined through discussion within the multidisciplinary research team. The guide explored

• Perceptions of current prevention programmes;
• Organisational and policy barriers;
• Facilitators of implementation;
• Stakeholder responsibilities;
• Recommendations for improving prevention strategies.

The complete interview guide is provided in Supporting Appendix S1.

Interviews were conducted in Persian, audio‐recorded with participants’ permission and transcribed verbatim immediately after completion.

Interview duration ranged from 40 to 60 min (median 50 min).

Field notes documenting contextual observations and researchers’ reflections were recorded immediately after each interview.

### 2.7. Data Saturation

Participant recruitment continued until meaning saturation was achieved.

Consistent with contemporary qualitative methodology, saturation was defined as the stage at which additional interviews no longer generated new concepts, categories or explanatory insights relevant to the study objectives.

Following identification of thematic redundancy, additional interviews were conducted to confirm analytical completeness before recruitment ceased.

### 2.8. Data Analysis

Data were analysed using Braun and Clarke’s six‐phase reflexive thematic analysis [32].

The analytical process comprised six iterative stages:

1. Familiarisation with the transcripts through repeated reading.
2. Generation of initial inductive codes.
3. Development of candidate categories and themes.
4. Review of themes against the complete dataset.
5. Refinement and naming of themes.
6. Preparation of the final thematic framework supported by representative quotations.

Coding was undertaken independently by Mehran Sardareh and Rona Bahreini, while Masoumeh Gholizadeh reviewed discrepancies and facilitated consensus meetings.

Data management was performed using MAXQDA (Version 10).

Analytical decisions were documented throughout the study using coding memos and an audit trail.

### 2.9. Research Team and Reflexivity

Because qualitative analysis is influenced by researchers’ perspectives, reflexivity was maintained throughout the study.

Before commencing data collection, members of the research team discussed their disciplinary backgrounds, prior assumptions regarding skin cancer prevention and potential influences on interpretation.

Throughout coding, regular reflexive meetings were held to challenge emerging interpretations and minimise researcher bias.

### 2.10. Trustworthiness

Trustworthiness was established according to the framework proposed by Guba and Lincoln [34].

 Credibility was enhanced through prolonged engagement with the data, investigator triangulation and synthesised member checking.
 Dependability was strengthened through maintenance of an audit trail documenting analytical decisions and coding revisions.
 Confirmability was promoted through independent coding, reflexive discussions and consensus meetings.
 Transferability was facilitated by providing detailed descriptions of participant characteristics, study context and analytical procedures to enable readers to judge applicability to other settings.

### 2.11. Member Checking

Following preliminary analysis, participants received a concise summary of the preliminary themes rather than full transcripts.

Participants were invited to comment on whether the interpretations accurately reflected their experiences.

Feedback confirmed the credibility of the thematic framework and did not require substantive modification of the final analysis.

### 2.12. Ethical Considerations

Ethical approval was obtained from the Ethics Committee of Tabriz University of Medical Sciences (Approval No. IR.TBZMED.REC.1399.243).

All participants provided written informed consent prior to participation.

Confidentiality was maintained by assigning anonymised identification codes to each participant, securely storing interview recordings and removing all identifying information from interview transcripts.

Participants were informed of their right to decline any question or withdraw from the study at any stage without consequence.

## 3. Results

A total of 18 purposively selected stakeholders participated in the qualitative study, including dermatologists, faculty members and healthcare professionals with substantial expertise in skin cancer prevention and management. Participants represented a broad spectrum of clinical, academic and public health perspectives, thereby ensuring maximum variation in professional experience and organisational roles. Their work experience ranged from 5 to 31 years, with a mean of 22 years, reflecting a highly experienced participant group capable of providing rich and information‐intensive data.

Among the participants, 11 (61.2%) were female and 7 (38.8%) were male. The majority were between 40 and 60 years of age (66.8%), while half of the participants held a medical specialty or fellowship degree. Dermatologists constituted the largest professional group (50%), followed by healthcare professionals (27.7%) and university faculty members (22.3%). Most participants (55.4%) had more than 15 years of professional experience. These characteristics ensured a comprehensive representation of stakeholders involved in clinical practice, education, public health and policy development (Table 1).

**TABLE 1:** Characteristics of participants in the qualitative study (18 people).

Variables		Frequency	Percent
Gender	Male	7	38.8%
	Female	11	61.2%

Age	Under 40 years	3	16.6%
	40–50 years	6	33.4%
	50–60 years	6	33.4%
	Over 60 years	3	16.6%

Job group	Dermatologists	9	50%
	Healthcare staff	5	27.7%
	Faculty member	4	22.3%

Education level	Bachelor’s/master’s	5	27.7%
	PhD	4	22.3%
	Specialty/fellowship	9	50%

Work experience (years)	5–10	3	16.6%
	10–15	5	27.7%
	Over 15	10	55.4%

Thematic analysis of the interview data generated a comprehensive policy‐oriented framework for skin cancer prevention. Following repeated familiarisation with the interview transcripts, meaningful units were coded inductively and subsequently organised into categories and subthemes through iterative comparison and consensus among the research team.

The five themes and eleven subthemes summarise the qualitative findings derived from the stakeholder interviews (Table 2). These findings were subsequently translated into potential intervention and implementation actions by the research team, with consideration of the organisational, healthcare, environmental and policy contexts identified across the dataset (Table 3). This analytical translation was used to inform the development of the stakeholder‐informed multilevel implementation framework. The resulting framework therefore distinguishes between participant‐derived qualitative findings and the authors’ subsequent synthesis of potential policy and implementation actions.

**TABLE 2:** Qualitative themes and subthemes identified from stakeholder interviews on skin cancer prevention.

Primary theme	Subtheme	Key qualitative findings
1. Public awareness and education	1.1. Targeted educational campaigns	Insufficient public knowledge regarding UV radiation, tanning risks, skin cancer risk factors, early signs and preventive behaviours; need for sustained and culturally appropriate education through mass media, social media and schools; emphasis on establishing sun‐protective behaviours during childhood.
	1.2. Community‐based outreach and training	Need for community‐based education and training on UV protection; use of community health workers as trusted intermediaries; importance of family‐centred education and community engagement in supporting preventive behaviours.

2. Environmental and structural modifications	2.1. Creating sun‐safe spaces	Need for shaded public and institutional environments, including parks, playgrounds, schools, recreational facilities and outdoor workplaces; environmental design should facilitate sun‐safe behaviours and reduce reliance on individual motivation.
	2.2. Adapting temporal exposure	Need to reduce exposure during periods of high UV intensity through flexible working hours, seasonal scheduling, staggered shifts and avoidance of peak UV periods, particularly for outdoor occupations.

3. Institutional and occupational policy	3.1. Workplace safety regulations	Occupational UV exposure was identified as an inadequately addressed workplace hazard; participants emphasised protective regulations, provision of personal protective equipment, occupational exposure monitoring, workplace risk assessment, industrial hygiene programmes and institutional accountability.
	3.2. Standards in educational and public settings	Need to incorporate sun protection education into school curricula and establish institutional policies supporting healthy lifestyles, preventive practices, shaded environments and visible prevention information in schools and other public settings.

4. Health system strengthening and access	4.1. Early detection and screening programmes	Need for organised screening and routine skin examinations for high‐risk groups; integration of prevention into primary healthcare and occupational health services; clear referral pathways and improved information systems to support follow‐up and continuity of care.
	4.2. Standardisation and capacity building	Need for evidence‐based clinical guidelines, standardised preventive protocols, continuing professional education and training in UV risk assessment, recognition of suspicious lesions, counselling and prevention; emphasis on multidisciplinary collaboration.

5. Holistic and intersectoral health promotion	5.1. Promoting general wellness	Promotion of healthy lifestyles as part of a broader prevention approach, including nutrition education and physical activity promotion.
	5.2. Comprehensive behavioural change initiatives	Need for integrated behavioural change strategies combining education with practical preventive behaviours, including sunscreen use and protective clothing.
	5.3. Cross‐sectoral environmental health	Recognition that skin cancer prevention is influenced by environmental and cross‐sectoral conditions and requires coordination across sectors to address broader environmental determinants of health.

**TABLE 3:** Translation of qualitative findings into proposed interventions, policy actions and expected outcomes for skin cancer prevention.

Qualitative theme	Proposed intervention/strategy	Policy or implementation action	Expected outcome
1. Public awareness and education	Targeted educational campaigns through mass media, social media and schools	Develop sustained, culturally appropriate public education programmes and integrate sun‐safety education into school curricula	Improved public knowledge, health literacy and adoption of preventive behaviours
1. Public awareness and education	Community‐based outreach and training	Train community health workers and strengthen family‐ and community‐centred prevention activities	Greater community engagement and sustained preventive practices
2. Environmental and structural modifications	Creation of sun‐safe spaces	Provide permanent shade structures in parks, playgrounds, schools, recreational facilities and outdoor workplaces	Reduced environmental UV exposure and improved opportunities for sun‐safe behaviour
2. Environmental and structural modifications	Adaptation of temporal exposure	Introduce flexible working hours, seasonal scheduling, staggered shifts and avoidance of peak UV periods where feasible	Reduced occupational and recreational UV exposure
3. Institutional and occupational policy	Workplace safety regulations	Establish occupational UV protection standards, provide protective equipment and shaded rest areas and incorporate UV exposure into workplace risk assessment and monitoring	Strengthened occupational protection and improved institutional accountability
3. Institutional and occupational policy	Standards in educational and public settings	Establish institutional policies supporting sun protection, healthy lifestyles, shaded environments and preventive education	Institutionalisation of preventive practices and improved continuity of prevention
4. Health system strengthening and access	Early detection and screening programmes	Integrate risk assessment, targeted screening, routine skin examinations and referral pathways into appropriate healthcare and occupational health services	Earlier identification of suspicious lesions and improved continuity of preventive care
4. Health system strengthening and access	Standardisation and capacity building	Develop evidence‐based clinical protocols and continuing professional education for healthcare providers	Improved provider competence, consistency of preventive practice and quality of care
5. Holistic and intersectoral health promotion	Promotion of general wellness	Integrate nutrition education, physical activity promotion and broader healthy‐lifestyle approaches into prevention programmes	Reinforcement of a broader prevention culture
5. Holistic and intersectoral health promotion	Comprehensive behavioural change initiatives	Combine education with promotion of sunscreen use, protective clothing and other practical sun‐protective behaviours	Improved adoption and maintenance of protective behaviours
5. Holistic and intersectoral health promotion	Cross‐sectoral environmental health	Strengthen coordination between health, education, occupational, environmental, municipal and other relevant sectors	More coordinated and sustainable prevention programmes
Cross‐cutting policy domain	Population‐level prevention and early detection	Align prevention, early detection, surveillance and service‐delivery strategies across relevant sectors	Improved population health outcomes, including earlier detection and potential reduction in disease burden
Cross‐cutting policy domain	Prevention‐oriented health system planning	Prioritise preventive services and efficient use of healthcare resources	Economic and efficiency gains, including potential reductions in avoidable healthcare expenditure and service pressure
Cross‐cutting policy domain	Institutionalisation and sustained governance	Establish long‐term, coordinated prevention structures supported by institutional commitment and intersectoral collaboration	Sustainable societal change, including a stronger prevention culture and more sustainable prevention programmes

Rather than functioning as isolated recommendations, these themes demonstrated substantial interrelationships, illustrating that effective skin cancer prevention requires coordinated interventions operating simultaneously at the individual, community, organisational, healthcare system and governmental levels. Collectively, these findings informed the development of a stakeholder‐informed implementation framework that links preventive actions with expected health, organisational and societal outcomes.

Although the interview guide explored policy, organisational, occupational and implementation dimensions of skin cancer prevention, the participant sample did not include specific representatives of patients, employers, insurers, municipalities or national‐level policymakers. Therefore, the resulting framework has been interpreted primarily as reflecting professional and health system perspectives.

### 3.1. Theme 1. Public Awareness and Education: Building the Foundation for Prevention

Public awareness and education emerged as the cornerstone of participants’ recommendations for improving skin cancer prevention. Stakeholders consistently described insufficient public knowledge regarding ultraviolet (UV) radiation, risk factors, early signs of skin cancer and preventive behaviours as one of the principal barriers to effective prevention. Participants emphasised that educational initiatives should extend beyond occasional awareness campaigns and become integrated within schools, healthcare services, community organisations and mass media.

Two complementary subthemes were identified: targeted educational campaigns and community‐based outreach and training.

Participants highlighted the need for sustained educational campaigns using multiple communication channels to increase awareness across different population groups. Mass media, social media platforms and school‐based educational programmes were regarded as particularly effective mechanisms for disseminating prevention messages.

Several participants emphasised that prevention should begin during childhood, arguing that healthy sun‐protective behaviours established early in life are more likely to persist into adulthood. Others stressed that educational materials should be culturally appropriate, easy to understand and tailored to different literacy levels.

Community engagement was considered equally important. Participants viewed community health workers as trusted intermediaries capable of translating scientific information into practical advice for families and local communities. Family‐centred education was repeatedly identified as an important strategy for promoting behavioural change and increasing acceptance of preventive practices.

#### 3.1.1. Interpretation

These findings suggest that stakeholders perceive education as substantially more than simple information dissemination. Instead, education was conceptualised as a continuous process of health literacy development supported through schools, healthcare providers, community organisations and public communication systems. Participants consistently argued that isolated awareness campaigns have limited long‐term effectiveness unless embedded within broader community‐based prevention programmes.

### 3.2. Theme 2. Environmental and Structural Modifications: Creating Supportive Environments

The second major theme highlighted the importance of modifying physical and organisational environments to facilitate sun‐safe behaviours. Participants repeatedly emphasised that prevention strategies should not rely exclusively on individual responsibility but should also create supportive environments that reduce unnecessary ultraviolet exposure.

Two interrelated subthemes were identified:

Stakeholders recommended increasing the availability of shaded public spaces, particularly in parks, playgrounds, schools, recreational facilities and outdoor workplaces. The provision of permanent shade structures was considered a practical intervention capable of substantially reducing cumulative UV exposure without requiring continuous behavioural effort from individuals.

Participants also emphasised the importance of modifying work schedules for outdoor occupations. Flexible working hours, seasonal scheduling, staggered shifts and avoidance of peak ultraviolet radiation periods were frequently proposed as practical organisational strategies. Such interventions were considered particularly relevant for construction workers, agricultural workers, municipal employees and other occupational groups with prolonged outdoor exposure.

Beyond physical infrastructure, participants highlighted the importance of environmental planning and urban design as components of comprehensive skin cancer prevention. They argued that healthy environments should be intentionally designed to support protective behaviours rather than relying solely on individual motivation.

#### 3.2.1. Interpretation

The findings indicate that stakeholders viewed environmental modification as an essential complement to educational interventions. While increasing knowledge may encourage behavioural change, supportive environments reduce structural barriers and make healthy choices easier to adopt and maintain. Participants therefore advocated for prevention strategies that combine behavioural education with environmental and organisational changes to achieve sustainable reductions in ultraviolet exposure.

### 3.3. Theme 3. Institutional and Occupational Policy: Establishing Protective Regulations

The third major theme highlighted the importance of institutional commitment and occupational policy in reducing skin cancer risk. Participants consistently argued that preventive efforts cannot rely solely on individual behavioural change and should instead be reinforced through organisational regulations, occupational health policy and mandatory safety standards. This theme comprised two subthemes: workplace safety regulations and standards in educational and public settings.

#### 3.3.1. Workplace Safety Regulations

Participants repeatedly emphasised that outdoor workers constitute one of the most vulnerable populations because of prolonged occupational exposure to ultraviolet radiation. Many stakeholders expressed concern that occupational UV exposure remains inadequately recognised as a workplace hazard and that preventive measures are inconsistently implemented across different sectors.

Several participants recommended the development of comprehensive workplace regulations requiring employers to provide appropriate personal protective equipment, including wide‐brimmed hats, protective clothing, sunscreen and access to shaded rest areas. Regular monitoring of occupational UV exposure and incorporation of skin cancer prevention into occupational health surveillance programmes were also identified as essential components of effective prevention.

Participants further highlighted that preventive regulations should be supported by industrial hygiene programmes and routine workplace inspections to ensure compliance with occupational safety standards. According to several stakeholders, institutional accountability is essential for sustaining preventive behaviours among employees working in high‐risk outdoor occupations.

#### 3.3.2. Standards in Educational and Public Settings

Beyond occupational environments, participants advocated for stronger policy standards within schools, universities, recreational facilities and other public institutions. They recommended incorporating sun protection education into national school curricula and developing institutional policies that promote healthy lifestyles and routine preventive practices.

Participants believed that educational institutions provide an ideal setting for establishing lifelong preventive behaviours because children and adolescents can acquire protective habits before long‐term ultraviolet exposure accumulates. Several stakeholders also suggested that public institutions should adopt formal policies encouraging the availability of shaded environments and visible educational materials promoting skin cancer prevention.

#### 3.3.3. Interpretation

Collectively, these findings demonstrate that stakeholders regarded institutional policies as essential mechanisms for translating prevention knowledge into routine practice. Rather than relying exclusively on voluntary behavioural change, participants consistently advocated for regulatory approaches capable of embedding preventive measures within organisational structures. Such policies were perceived as promoting equity by ensuring that preventive opportunities are available regardless of individual socioeconomic status or personal motivation.

### 3.4. Theme 4. Health System Strengthening and Access: Enhancing Preventive Capacity

Strengthening the healthcare system emerged as another major theme during data analysis. Participants consistently emphasised that primary healthcare services represent an underutilised platform for improving skin cancer prevention through early detection, systematic risk assessment, professional education and evidence‐based clinical practice. Two closely related subthemes were identified: early detection and screening programmes and standardisation and capacity building.

#### 3.4.1. Early Detection and Screening Programmes

Participants highlighted the importance of establishing organised screening programmes for individuals at increased risk of skin cancer, including outdoor workers, older adults, immunocompromised individuals and people with a family history of melanoma. Stakeholders argued that early diagnosis substantially improves treatment outcomes while reducing healthcare costs associated with advanced disease management.

Several participants recommended integrating routine skin examinations into primary healthcare services and occupational health programmes. Others emphasised that screening initiatives should be accompanied by clear referral pathways to ensure timely specialist assessment of suspicious lesions. Participants also proposed strengthening electronic health information systems to facilitate follow‐up and continuity of care for high‐risk individuals.

#### 3.4.2. Standardisation and Capacity Building

Another important finding concerned the need for standardised clinical guidelines and continuous professional development. Participants reported considerable variation in preventive practices among healthcare providers and recommended developing national evidence‐based protocols to improve consistency of care.

Training programmes focusing on ultraviolet radiation, risk assessment, early recognition of suspicious skin lesions, and patient counselling were considered essential for healthcare professionals working in primary care, occupational health and dermatology services. Participants further emphasised that improving provider competence would increase public confidence in prevention programmes and facilitate earlier identification of skin cancers.

Several stakeholders also highlighted the importance of multidisciplinary collaboration among dermatologists, general practitioners, nurses, public health professionals and occupational physicians to strengthen continuity of preventive services across different levels of the healthcare system.

#### 3.4.3. Interpretation

These findings indicate that participants viewed healthcare systems not merely as providers of treatment but as central actors in disease prevention. Strengthening primary healthcare infrastructure, improving professional competence, implementing evidence‐based clinical protocols and expanding organised screening programmes were consistently regarded as essential components of an integrated prevention strategy. The convergence of these recommendations suggests that sustainable skin cancer prevention requires systematic investment in healthcare capacity alongside broader community and policy interventions.

### 3.5. Theme 5. Multisectoral Collaboration and Policy Integration: Developing a Sustainable Prevention Framework

The final theme underscored the necessity of coordinated action across multiple sectors to establish a sustainable and comprehensive skin cancer prevention strategy. Participants consistently emphasised that no single institution or professional group possesses sufficient capacity to effectively address the growing burden of skin cancer. Instead, successful prevention requires collaboration among healthcare organisations, governmental agencies, educational institutions, employers, media organisations, municipalities and community groups.

Three interconnected subthemes emerged within this theme: intersectoral collaboration, governmental leadership and policy commitment, and sustainable monitoring and evaluation.

#### 3.5.1. Intersectoral Collaboration

Participants described fragmentation of existing prevention initiatives as one of the principal barriers to effective implementation. Although numerous organisations currently conduct educational activities or preventive programmes, these efforts frequently occur independently with limited communication, coordination or resource sharing.

Several stakeholders emphasised that stronger collaboration between healthcare providers, educational institutions, occupational health services, municipalities and media organisations would substantially improve programme efficiency and population coverage. Participants suggested establishing national coordinating committees responsible for integrating prevention activities across sectors and facilitating information exchange among relevant stakeholders.

In addition, participants highlighted the importance of involving nongovernmental organisations, community leaders and patient advocacy groups to increase community engagement and improve public acceptance of prevention programmes.

#### 3.5.2. Interpretation

These findings suggest that stakeholders considered collaboration itself to be a preventive intervention. Rather than viewing prevention as the responsibility of individual organisations, participants consistently advocated for integrated governance structures capable of aligning institutional priorities, coordinating available resources and promoting long‐term sustainability.

#### 3.5.3. Governmental Leadership and Policy Commitment

Participants consistently identified governmental commitment as the fundamental prerequisite for successful implementation of skin cancer prevention programmes. They argued that sustainable prevention requires national legislation, adequate financial investment, clear strategic priorities and long‐term policy support.

Several stakeholders emphasised that prevention programmes frequently fail because financial resources remain focused primarily on treatment rather than prevention. Participants therefore recommended increasing governmental investment in public education, occupational protection, environmental modification and research supporting evidence‐based prevention policies.

Furthermore, participants highlighted the importance of incorporating skin cancer prevention into national public health agendas and noncommunicable disease strategies. Establishing measurable national indicators, strengthening surveillance systems and ensuring accountability among implementing organisations were also regarded as essential governmental responsibilities.

#### 3.5.4. Interpretation

Governmental leadership emerged not only as a source of financial support but also as a critical mechanism for ensuring policy continuity, institutional accountability and equitable access to preventive services. Participants believed that coordinated national leadership would facilitate implementation of comprehensive prevention strategies across healthcare, education, occupational health and environmental sectors.

#### 3.5.5. Sustainable Monitoring and Evaluation

Participants recognised that prevention programmes should be continuously evaluated to determine their effectiveness, identify implementation challenges and guide future policy refinement.

Suggested evaluation indicators included

• Population awareness of ultraviolet radiation risks;
• Sunscreen utilisation rates;
• Adoption of sun‐protective behaviours;
• Occupational compliance with protective regulations;
• Participation in screening programmes;
• Incidence of advanced‐stage skin cancer;
• Accessibility of preventive healthcare services.

Stakeholders also recommended establishing national monitoring systems capable of tracking programme performance over time and supporting evidence‐informed policy decisions.

#### 3.5.6. Interpretation

These findings demonstrate that participants regarded monitoring and evaluation as essential components of implementation rather than activities performed only after programme completion. Continuous assessment was viewed as necessary to ensure programme quality, optimise resource allocation and promote long‐term sustainability.

#### 3.5.7. Integration of Findings

Across all interviews, stakeholders consistently emphasised that skin cancer prevention requires coordinated interventions operating simultaneously at multiple levels of society. Educational initiatives alone were considered insufficient unless supported by environmental modifications, occupational protection, healthcare system strengthening, governmental commitment and effective intersectoral collaboration.

The five themes identified through thematic analysis collectively illustrate that prevention should be conceptualised as an integrated public health system rather than a collection of isolated interventions. The interaction among educational, organisational, healthcare, environmental and policy‐related factors formed the basis for the stakeholder‐informed implementation framework developed in this study (Figure 1).

**FIGURE 1:**
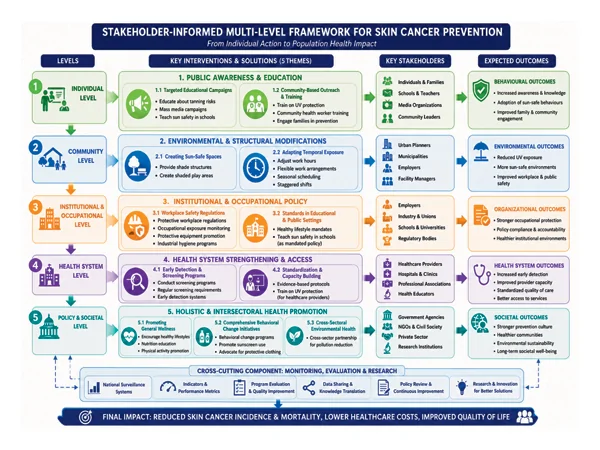
Stakeholder‐informed multilevel framework for skin cancer prevention. Developed by the authors based on the qualitative findings and thematic synthesis of the present study.

This framework demonstrates how interventions at different levels reinforce one another to facilitate behavioural change, improve healthcare delivery, strengthen institutional capacity and ultimately reduce the burden of skin cancer at the population level.

## 4. Discussion

### 4.1. Main Findings and Contribution of the Study

This qualitative study explored stakeholders’ perspectives regarding barriers, priorities and implementation strategies for improving skin cancer prevention. Through thematic analysis, five interconnected domains were identified: (1) public awareness and education, (2) environmental and structural modifications, (3) institutional and occupational policy, (4) health system strengthening and access and (5) multisectoral collaboration and policy integration. Collectively, these findings informed the development of a stakeholder‐informed multilevel framework that conceptualises skin cancer prevention as a coordinated public health strategy rather than a series of isolated individual behaviours.

The central contribution of this study is the shift from a predominantly individual‐focused prevention approach toward a broader system‐based perspective. Although previous research has extensively demonstrated the effectiveness of sun‐protective behaviours, sunscreen use, early detection and risk communication interventions, increasing evidence indicates that sustainable skin cancer prevention requires addressing broader determinants, including environmental conditions, healthcare organisation, social context and policy‐level influences [35, 36].

The findings of the present study extend previous evidence by demonstrating that successful prevention depends not only on the availability of effective interventions but also on the capacity of healthcare systems, organisations and communities to implement and sustain these strategies. This perspective is consistent with contemporary implementation science frameworks, which emphasise the importance of contextual factors, stakeholder engagement, organisational readiness and adaptive implementation processes in translating evidence‐based interventions into real‐world practice [14].

By incorporating perspectives from professionals involved in clinical care, public health, research and policy development, this study provides insights into the structural and organisational barriers influencing prevention implementation. Multistakeholder approaches are increasingly recognised as essential for addressing complex public health challenges, where outcomes are shaped by interactions between individual behaviours, social environments, institutional structures and governmental policies [37].

### 4.2. Moving Beyond Individual Behaviour Change: The Role of Health Literacy and Public Engagement

One of the strongest findings of this study was the recognition that insufficient public awareness remains a major obstacle to effective skin cancer prevention. Participants emphasised that knowledge gaps regarding ultraviolet (UV) radiation exposure, tanning behaviours, early signs of skin cancer and protective practices continue to limit prevention efforts.

These findings are consistent with previous evidence demonstrating that health literacy plays a fundamental role in individuals’ ability to obtain, understand, evaluate and use health information for preventive decision‐making [38]. Limited health literacy may reduce individuals’ capacity to accurately perceive cancer risk, interpret prevention recommendations and adopt sustained protective behaviours.

However, the findings of this study extend previous research by highlighting that awareness alone is insufficient to generate meaningful and long‐lasting behavioural change. Contemporary behavioural theories indicate that health behaviours are influenced by multiple interacting determinants, including individual motivation, social norms, environmental opportunities, perceived risk and accessibility of preventive resources [39].

Participants therefore supported a transition from traditional information‐based campaigns toward comprehensive health literacy strategies that integrate education with supportive environments and institutional reinforcement. Previous research suggests that multicomponent interventions combining education, community engagement, environmental modification and policy support are more effective than isolated educational approaches in improving sun‐protective behaviours [40].

The emphasis placed on childhood education is particularly important. Early‐life UV exposure and sunburns during childhood and adolescence are strongly associated with increased melanoma risk later in life, making early prevention an important component of population‐based cancer control strategies [41]. Schools provide an ideal setting for establishing sun‐safe behaviours because they allow repeated exposure to preventive messages and integration of protective practices into daily routines.

Evidence from school‐based skin cancer prevention interventions suggests that educational programmes are more likely to achieve meaningful changes in sun‐protective knowledge and behaviours when they incorporate multiple components and are supported by the school environment and institutional practices. School‐level implementation support involving teachers and other school personnel can further strengthen the adoption of sun‐safety practices, while environmental measures such as access to shade can complement educational approaches by facilitating protective behaviour [42]. Therefore, incorporating skin cancer prevention into educational curricula may represent a sustainable approach for reducing future disease burden.

### 4.3. Environmental and Structural Approaches: Creating Conditions That Enable Prevention

A major finding of this study was that participants considered environmental modification essential for sustainable skin cancer prevention. Rather than placing responsibility exclusively on individuals, stakeholders emphasised that communities, organisations and policymakers should create environments that make protective behaviours easier, accessible and socially acceptable.

This perspective aligns with ecological models of health promotion, which conceptualise health behaviours as the result of interactions between individuals and multiple levels of influence, including interpersonal relationships, organisational settings, community structures and public policies [24]. In the context of skin cancer prevention, individuals may understand the importance of sun protection but remain exposed because of environmental limitations, occupational requirements, inadequate infrastructure or limited access to protective resources.

Participants particularly highlighted the importance of shaded public spaces, modification of outdoor activities during periods of high UV radiation and improved access to protective facilities. These findings are consistent with previous evidence demonstrating that environmental interventions can reduce UV exposure by modifying exposure conditions rather than relying solely on continuous individual decision‐making [43].

Public shade provision represents an important example of a population‐level intervention. Unlike behavioural interventions that require repeated personal motivation, structural interventions can provide passive protection for large groups, including children, outdoor workers and socially vulnerable populations. Studies evaluating community‐based shade interventions have suggested that environmental modifications can improve sun protection practices and reduce cumulative UV exposure [44].

Importantly, environmental approaches may also contribute to reducing health inequalities. Prevention strategies that depend primarily on individual responsibility may disproportionately affect populations with fewer socioeconomic resources or limited control over their exposure environments. Outdoor workers, low‐income communities and individuals living in areas with insufficient public infrastructure may experience greater barriers to adopting protective behaviours [45].

Therefore, structural interventions should not be considered merely supportive measures but rather essential components of equitable cancer prevention strategies. A system‐based approach requires governments, municipalities, educational institutions and workplaces to share responsibility for creating environments that facilitate prevention.

### 4.4. Occupational Protection and Institutional Responsibility

Occupational UV exposure emerged as a critical area requiring greater policy attention. Participants consistently identified outdoor workers as a high‐risk population and expressed concerns regarding insufficient recognition of solar UV radiation as an occupational hazard.

Previous epidemiological studies have demonstrated that cumulative occupational exposure to solar UV radiation significantly increases the risk of keratinocyte carcinomas and contributes substantially to the global burden of nonmelanoma skin cancers, particularly among agricultural workers, construction workers, fishermen and other outdoor occupations [46, 47].

Despite strong scientific evidence, occupational skin cancer prevention remains inconsistently implemented in many countries. Previous research has identified several barriers, including limited employer awareness, inadequate regulatory enforcement, competing workplace priorities and the perception that sun exposure is an unavoidable aspect of outdoor employment [48].

The findings of this study highlight the need to redefine prevention responsibility. Effective occupational protection cannot depend solely on workers’ personal choices; instead, employers, occupational health services and regulatory authorities must assume active responsibility. Similar approaches have been successfully applied in other occupational health areas where environmental risks require institutional control rather than individual adaptation [49].

Strategies such as mandatory access to protective clothing, availability of shade structures, scheduling outdoor activities outside periods of maximum UV intensity, worker education and routine workplace risk assessment should become integrated components of occupational safety programmes [50].

This finding has important policy implications because workplace interventions provide repeated opportunities to reach populations with prolonged UV exposure. Integrating skin cancer prevention into occupational health regulations may therefore represent one of the most impactful strategies for reducing preventable disease burden among high‐risk populations.

### 4.5. Strengthening Healthcare Systems: From Treatment‐Oriented Care to Prevention‐Oriented Models

Participants identified healthcare systems as central actors in strengthening skin cancer prevention, particularly through early detection, professional education, risk assessment and integration of preventive services into routine healthcare delivery.

Although advances in systemic therapies, including immune checkpoint inhibitors and targeted therapies, have substantially improved outcomes for patients with advanced melanoma, prevention and early diagnosis remain fundamental because they reduce disease burden before patients require complex and costly treatments [51]. Modern cancer control frameworks increasingly emphasise that therapeutic advances should be accompanied by strong investments in prevention and early detection programmes [52].

The findings suggest that primary healthcare represents an underutilised platform for skin cancer prevention. Primary care professionals, including physicians, nurses and community healthcare providers, frequently interact with individuals at risk and are therefore well positioned to provide preventive counselling, identify high‐risk individuals and facilitate early referral [53].

However, participants highlighted important barriers, including inadequate professional training, limited awareness among healthcare providers, inconsistent clinical guidelines and weak coordination between primary care and specialist dermatology services. Previous studies have similarly demonstrated that healthcare professionals may lack confidence in performing skin cancer risk assessment and counselling because of limited training opportunities and competing clinical priorities [54].

Addressing these challenges requires integration of skin cancer prevention into undergraduate medical education, continuing professional development programmes, clinical guidelines and healthcare quality improvement initiatives. Structured risk assessment tools, digital decision‐support systems and improved referral pathways may further enhance preventive capacity within primary healthcare settings [55].

The integration of prevention into healthcare systems represents a fundamental transition from reactive disease management toward proactive population health management. Such an approach reflects the broader principles of preventive medicine, where healthcare systems actively identify and modify risk factors before disease development occurs [56].

### 4.6. Stakeholder Collaboration and the Need for Policy‐Level Governance

Perhaps the most important overarching finding of this study was the recognition that skin cancer prevention requires coordinated action across multiple sectors. Participants consistently rejected fragmented approaches and advocated for governance structures capable of aligning healthcare providers, policymakers, educational institutions, employers, municipalities and communities.

This finding supports contemporary implementation science perspectives, which emphasise that successful public health interventions depend not only on intervention effectiveness but also on contextual adaptation, stakeholder engagement, organisational capacity and sustained political commitment [14]. Complex prevention challenges such as skin cancer cannot be effectively addressed through isolated behavioural interventions because determinants of exposure and risk are distributed across multiple social and institutional levels.

Previous prevention initiatives have frequently evaluated individual interventions, such as sunscreen promotion or educational campaigns, without sufficiently considering the broader systems in which implementation occurs. Systems‐based approaches argue that sustainable health improvements require understanding interactions among policies, organisations, environments and individual behaviours [57]. The multilevel framework developed in this study contributes to this perspective by demonstrating how different prevention components interact across healthcare, community, occupational, educational and policy domains.

Participants identified governmental leadership as a critical enabling factor for sustainable prevention. Previous international experiences indicate that successful cancer prevention programmes require political commitment, dedicated resources, surveillance systems, regulatory mechanisms and long‐term strategic planning [58]. Without these elements, prevention initiatives may remain fragmented, short‐term and dependent on temporary projects rather than becoming integrated components of national health policy.

Therefore, skin cancer prevention should be incorporated into broader national cancer control strategies and noncommunicable disease prevention programmes. Establishing population‐based UV surveillance systems, strengthening occupational protection regulations, integrating prevention into primary healthcare and supporting community‐based initiatives represent essential steps toward sustainable disease reduction [59].

### 4.7. Implications for Research, Policy and Practice

The findings of this study provide several important implications for future research, policy development and clinical practice.

First, policymakers should develop integrated prevention strategies that combine public education, environmental modification, occupational protection, healthcare strengthening and governance mechanisms. Evidence from other public health fields demonstrates that multilevel interventions addressing multiple determinants are more likely to achieve sustained behavioural and population‐level outcomes compared with single‐component interventions [60].

Second, healthcare systems should expand the preventive role of primary care by incorporating structured risk assessment, preventive counselling, early detection pathways and professional education into routine healthcare services. Strengthening primary care capacity may improve early identification of high‐risk individuals and reduce delays in diagnosis.

Third, employers and regulatory organisations should formally recognise occupational UV exposure as an important workplace health issue. Mandatory workplace protection policies, rather than voluntary recommendations alone, are required to ensure consistent protection of outdoor workers.

Fourth, future research should evaluate the effectiveness, feasibility, acceptability, sustainability and cost‐effectiveness of multilevel skin cancer prevention frameworks across different cultural, socioeconomic and healthcare contexts. Implementation research approaches, including hybrid effectiveness implementation studies, may provide valuable evidence regarding how prevention interventions can be successfully integrated into real‐world settings [61].

The stakeholder‐informed framework proposed in this study provides a practical foundation for designing, implementing and evaluating comprehensive skin cancer prevention programmes. Unlike approaches focusing on single preventive behaviours, this framework conceptualises prevention as a dynamic system requiring interaction between individuals, organisations, healthcare structures, environmental conditions and policy environments.

### 4.8. Strengths and Limitations

This study has several strengths. First, the inclusion of diverse stakeholders provided comprehensive perspectives from clinical, organisational, public health and policy domains. This multidisciplinary approach enhanced understanding of barriers and facilitators that may remain unidentified when prevention is examined from a single professional perspective.

Second, the qualitative methodology enabled exploration of complex organisational and contextual factors influencing prevention implementation. Such factors are often difficult to capture through quantitative approaches alone because they involve perceptions, experiences, institutional cultures and social processes.

Third, the development of a multilevel stakeholder‐informed framework represents an important contribution by translating qualitative findings into actionable recommendations for policy and practice. This approach may assist decision‐makers in designing prevention programmes that are better aligned with local needs and implementation realities.

However, several limitations should also be acknowledged.

First, participants were recruited from a specific healthcare and academic context, which may limit transferability to other regions with different healthcare structures, cultural characteristics and policy environments. Nevertheless, detailed descriptions of the study context, participant characteristics and analytical processes were provided to enable readers to assess the applicability of findings to other settings, consistent with qualitative research principles of transferability [62].

Second, although maximum variation sampling was employed, some stakeholder groups, including members of the general public, employers, insurance organisations and national‐level policymakers, may have been underrepresented. Future studies incorporating broader stakeholder groups may provide additional perspectives and further refine the proposed framework.

Third, qualitative findings reflect participants’ perceptions and professional experiences and should therefore be interpreted within this context. Future quantitative and mixed‐method implementation evaluations are needed to examine the effectiveness, feasibility, economic impact and sustainability of proposed prevention strategies.

An important limitation concerns the composition of the stakeholder sample. Although maximum variation sampling was used across professional backgrounds and organisational roles, the final sample was predominantly composed of dermatologists, healthcare professionals and university faculty members. Perspectives from patients, employers, insurers, municipalities and national‐level policymakers were not directly represented. Consequently, some contextual factors related to patient preferences, workplace implementation, financing, municipal infrastructure, insurance mechanisms and national policy execution may not have been fully captured. The proposed framework should therefore be considered stakeholder‐informed but not fully representative of all actors involved in skin cancer prevention. Its transferability and implementation relevance across these stakeholder groups require further evaluation through broader, multisectoral engagement and subsequent implementation studies.

Finally, although reflexivity procedures and investigator triangulation were used to enhance methodological rigor, researcher interpretation remains an inherent component of qualitative analysis. Future studies involving independent research teams from different geographical and institutional contexts may further strengthen the external validity of findings.

## 5. Conclusion

Effective skin cancer prevention requires a comprehensive transition from isolated behavioural interventions toward integrated, multilevel public health strategies. This study demonstrates that successful prevention depends on strengthening health literacy, creating supportive environments, implementing occupational protections, enhancing healthcare capacity and establishing coordinated policy leadership.

The stakeholder‐informed framework developed in this study provides a practical roadmap for translating evidence into sustainable prevention action. Future efforts should focus on implementing and evaluating such integrated approaches to reduce skin cancer incidence, improve population health outcomes and decrease the long‐term healthcare burden associated with preventable disease.

## Author Contributions

Masoumeh Gholizadeh and Rona Bahreini designed the study, analysed the data and wrote the initial draft of the study. Mehran Sardareh, Amin Ghanbari and Safoura Pourreza participated in data analysis and drafting of the initial draft of the study. Masoumeh Gholizadeh designed the study, searched databases, screened studies, extracted and analysed data and wrote the first draft of the study and acted as supervisor. All authors reviewed the manuscript.

## Funding

The research protocol was approved and supported by Hematology and Oncology Research Center, Tabriz University of Medical Sciences (Grant No. 64221).

## Ethics Statement

The present study has been approved by the Ethics Committee of Tabriz University of Medical Sciences with the ethics code IR.TBZMED.REC.1399.243. Informed consent was obtained from the participants, and the principles of confidentiality were observed in the characteristics and information of the individuals. The individuals were assured that the results of the study would only be used for the purposes of the study and would not be used in other cases, and each individual was allowed to leave the study at any stage of the study without harm or loss.

## Consent

Please see the Ethics Statement.

## Conflicts of Interest

The authors declare no conflicts of interest.

## Supporting Information

Additional supporting information can be found online in the Supporting Information section.

## Supporting information


**Supporting Information** Interview questions from experts.

## Data Availability

All data generated or analysed during this study are included in this published article.
