# Supplementary material for: Developing a Stakeholder‐Informed Multilevel Framework for Skin Cancer Prevention: A Qualitative Study of Barriers, Policy Priorities and Implementation Strategies
Source: J Skin Cancer. 2026 Nov 8;2026:6064093. doi: 10.1155/jskc/6064093 (PMC13745987; doi:10.1155/jskc/6064093)
Supplement: Supplementary file 1 [file JSKC-2026-6064093-s001.docx]

**Dear Expert and Colleague,**

Greetings and respect.

This guide has been designed to determine your views and attitudes regarding the provision of policy solutions for skin cancer prevention. We kindly request your cooperation in answering the questions so that we may understand your perspective on policy solutions for skin cancer prevention. Additionally, with your permission, your views will be audio-recorded. We assure you that you may withdraw from the study at any time if you wish. It is important to note that this guide has been designed without personal or employment identifiers, and all information will be treated as completely confidential and used solely for research purposes at Tabriz University of Medical Sciences. We sincerely thank you in advance for your valuable cooperation.

**Demographic Characteristics and Background Information**

- **Gender:** Male □ Female □
- **Age:** ............ years
- **Marital Status:** Single □ Married □
- **Work Experience:** ............ years
- **Education Level:** Bachelor's □ Master's □ PhD □ Professional Doctorate □ Medical Specialty □
- **Field of Latest Degree:** ................................................
- **Organizational Position:** ................................................
- **Panel Date:** ............ **Panel Location:** ............

**Questions**

1. What might skin cancer prevention policies include?
2. What are the target groups and implementation settings for these policies? (e.g., schools and students)
3. What factors can be influential in the development, implementation, and execution of skin cancer prevention policies?
4. What are the benefits of properly developing and implementing skin cancer prevention policies?
5. Is there anything else regarding this topic that you would like to discuss?
